# Hyperspectral Imaging for the Detection of Glioblastoma Tumor Cells in H&E Slides Using Convolutional Neural Networks

**DOI:** 10.3390/s20071911

**Published:** 2020-03-30

**Authors:** Samuel Ortega, Martin Halicek, Himar Fabelo, Rafael Camacho, María de la Luz Plaza, Fred Godtliebsen, Gustavo M. Callicó, Baowei Fei

**Affiliations:** 1 Quantitative Bioimaging Laboratory, Department of Bioengineering, The University of Texas at Dallas, Richardson, TX 75080, USA; mth180000@utdallas.edu; 2 Institute for Applied Microelectronics (IUMA), University of Las Palmas de Gran Canaria (ULPGC), 35017 Las Palmas de Gran Canaria, Spain; hfabelo@iuma.ulpgc.es (H.F.); gustavo@iuma.ulpgc.es (G.M.C.); 3 Department of Biomedical Engineering, Emory University and Georgia Institute of Technology, 1841 Clifton Road NE, Atlanta, GA 30329, USA; 4 Department of Pathological Anatomy, University Hospital Doctor Negrin of Gran Canaria, Barranco de la Ballena s/n, 35010 Las Palmas de Gran Canaria, Spain; rcamgal@gobiernodecanarias.org (R.C.); mplaperb@gobiernodecanarias.org (M.d.l.L.P.); 5 Department of Mathematics and Statistics, UiT The Artic, University of Norway, Hansine Hansens veg 18, 9019 Tromsø, Norway; fred.godtliebsen@uit.no; 6 Advanced Imaging Research Center, University of Texas Southwestern Medical Center, 5323 Harry Hine Blvd, Dallas, TX 75390, USA; 7 Department of Radiology, University of Texas Southwestern Medical Center, 5323 Harry Hine Blvd, Dallas, TX 75390, USA

**Keywords:** hyperspectral imaging, medical optics and biotechnology, optical pathology, convolutional neural networks, tissue diagnostics, tissue characterization, glioblastoma

## Abstract

Hyperspectral imaging (HSI) technology has demonstrated potential to provide useful information about the chemical composition of tissue and its morphological features in a single image modality. Deep learning (DL) techniques have demonstrated the ability of automatic feature extraction from data for a successful classification. In this study, we exploit HSI and DL for the automatic differentiation of glioblastoma (GB) and non-tumor tissue on hematoxylin and eosin (H&E) stained histological slides of human brain tissue. GB detection is a challenging application, showing high heterogeneity in the cellular morphology across different patients. We employed an HSI microscope, with a spectral range from 400 to 1000 nm, to collect 517 HS cubes from 13 GB patients using 20× magnification. Using a convolutional neural network (CNN), we were able to automatically detect GB within the pathological slides, achieving average sensitivity and specificity values of 88% and 77%, respectively, representing an improvement of 7% and 8% respectively, as compared to the results obtained using RGB (red, green, and blue) images. This study demonstrates that the combination of hyperspectral microscopic imaging and deep learning is a promising tool for future computational pathologies.

## 1. Introduction

Traditional diagnosis of histological samples is based on manual examination of the morphological features of specimens by skilled pathologists. In recent years, the use of computer-aided technologies for aiding in this task is an emerging trend, with the main goal to reduce the intra and inter-observer variability [1]. Such technologies are intended to improve the diagnosis of pathological slides to be more reproducible, increase objectivity, and save time in the routine examination of samples [2,3]. Although most of the research carried out in computational pathology has been in the context of RGB (red, green, and blue) image analysis [4,5,6,7], hyperspectral (HS) and multispectral imaging are shown as promising technologies to aid in the histopathological analysis of samples.

Hyperspectral imaging (HSI) is a technology capable of capturing both the spatial and spectral features of the materials that are imaged. Recently, this technology has proven to provide advantages in the diagnosis of different types of diseases [8,9,10]. In the field of histopathological analysis, this technology has been used for different applications, such as the visualization of multiple biological markers within a single tissue specimen with inmunohistochemistry [11,12,13], the digital staining of samples [14,15], or diagnosis.

The analysis of HS images is usually performed in combination with machine learning approaches [16]. Traditionally, feature-based methods are used, such as supervised classifiers. Awan et al. performed automatic classification of colorectal tumor samples identifying four types of tissues: normal, tumor, hyperplastic polyp, and tubular adenoma with low-grade dysplasia. Using different types of feature extraction and band selection methods followed by support vector machines (SVM) classification, the authors found that the use of a higher number of spectral bands improved the classification accuracy [17]. Wang et al. analyzed hematoxylin and eosin (H&E) skin samples to facilitate the diagnosis of melanomas. The authors proposed a customized spatial-spectral classification method, which provided an accurate identification of melanoma and melanocytes with high specificity and sensitivity [18]. Ishikawa et al. presented a method for pancreatic tumor cell identification using HSI. They first proposed a method to remove the influence of the staining in the HS data, and then they applied SVM classification [19].

Although these authors have proven the feasibility of the feature learning methods for the diagnosis of histopathological samples using HS information, the performance of these approaches may be improved by using deep learning (DL) schemes. DL approaches automatically learn from the data in which features are optimal for classification, potentially outperforming handcrafted features [20]. In the case of HS images, both the spatial and spectral features are exploited simultaneously. Recently, only a few researchers employed DL for the classification of HS images for histopathological applications. Malon et al. proposed the use of a convolutional neural network (CNN) for the detection of mitotic cells within breast cancer specimens [21]. Haj-Hassan et al. also used CNNs for the classification of colorectal cancer tissue, showing performance improvements compared to traditional feature learning approaches [22].

In this paper, we propose the use of CNNs for the classification of hematoxylin and eosin (H&E) stained brain tumor samples. Specifically, the main goal of this work was to differentiate between high-grade gliomas (i.e., glioblastoma (GB)) and non-tumor tissue. In a previous study, we presented a feature learning approach for this type of disease [23]. Although such research was shown as a useful proof-of-concept on the possibilities of HS for histopathological analysis of GB, it presented some limitations, such as poor spatial and spectral resolution, and the lack of a rigorous experimental design. In this work, the image quality and spectral range have been significantly improved, resulting in a more appropriate experimental design for realistic clinical applications.

## 2. Materials and Methods

### 2.1. Acquisition System

**Figure 1:**
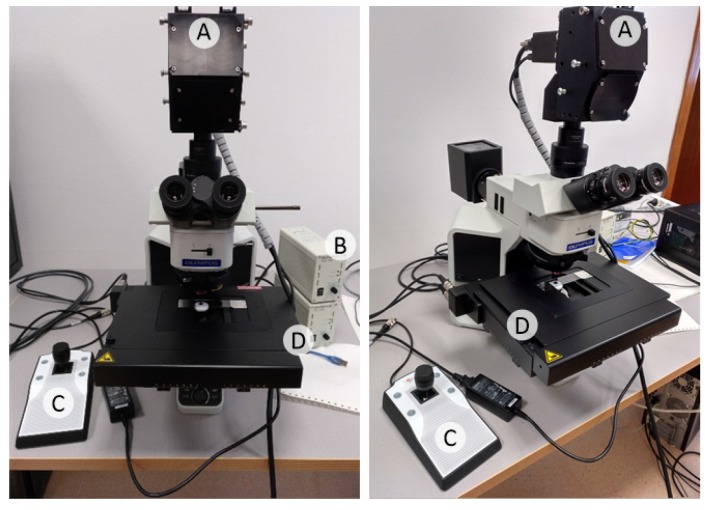
Microscopic hyperspectral (HS) acquisition system. (**A**) HS camera. (**B**) Halogen light source. (**C**) Positioning joystick. (**D**) XY linear stage.

The instrumentation employed in this study consists of an HS camera coupled to a conventional light microscope (Figure 1). The microscope is an Olympus BX-53 (Olympus, Tokyo, Japan). The HS camera is a Hyperspec^®^ VNIR A-Series from HeadWall Photonics (Fitchburg, MA, USA), which is based on an imaging spectrometer coupled to a CCD (Charge-Coupled Device) sensor, the Adimec-1000m (Adimec, Eindhoven, Netherlands). This HS system works in the visual and near-infrared (VNIR) spectral range from 400 to 1000 nm with a spectral resolution of 2.8 nm, sampling 826 spectral channels and 1004 spatial pixels. The push-broom camera performs spatial scanning to acquire an HS cube with a mechanical stage (SCAN, Märzhäuser, Wetzlar, Germany) attached to the microscope, which provides accurate movement of the specimens. The objective lenses are from the LMPLFLN family (Olympus, Tokyo, Japan), which are optimized for infra-red (IR) observations. The light source is a 12 V, 100 W halogen lamp.

To ensure high quality acquisitions, the methodology proposed in a previous research work to maximize the quality of HS images acquired with a push-broom microscope [24] was followed. This methodology includes the optimal speed determination of the scanning, a dynamic range configuration, an appropriate alignment, and the correct focusing procedure. We developed custom software for synchronizing the scanning movement and the camera acquisition. Although we are not focused on collecting a whole-slide HS image of the specimens, the software was developed to allow the acquisition of consecutive HS cubes in a row to save time in the acquisition of the images, thus reducing the human intervention in the process. Due to the challenges imposed by the high dimensionality of the HS images, we decided to collect images with a spatial size of 800 lines, producing HS cubes of 800 × 1004 × 826, i.e., number of lines × number of rows × number of bands.

### 2.2. GB Histological Samples

**Figure 2:**
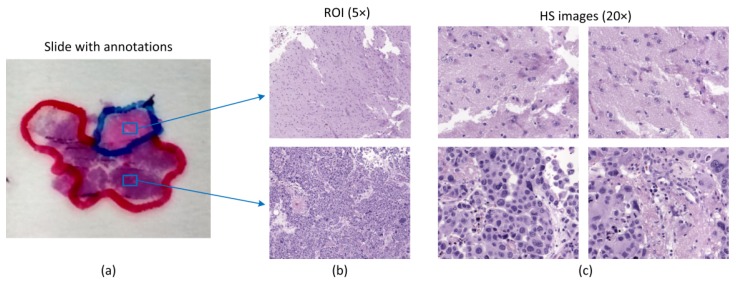
Pathological samples used in this study. (**a**) Macroscopic annotations performed in pathological slides after diagnosis. Blue squares denote regions of interest (ROIs) within annotations; (**b**) ROIs from (**a**) shown at 5×; (**c**) Examples of HS images used in this study for classification (imaged at 20×).

The specimens investigated in this research work consist of human biopsies extracted during brain tumor resection procedures. The pathological slides in this study were processed and analyzed by the Pathological Anatomy Department of the University Hospital Doctor Negrín at Las Palmas of Gran Canaria (Spain). The study protocol and consent procedures were approved by the Comité Ético de Investigación Clínica-Comité de Ética en la Investigación (CEIC/CEI) of the same hospital. After the resection, the samples were dehydrated and embedded in paraffin blocks. The blocks were then mounted in microtomes and sliced in 4 µm thick slices. Finally, the slices were rehydrated and stained with H&E. After routine examination of the samples, every sample was diagnosed by pathologists as GB, according to the World Health Organization (WHO) classification of tumors of the nervous system [25]. After the pathologist confirmed the GB diagnosis, macroscopic annotations of the GB locations were made on the physical glass slides using a marker-pen. Non-tumor areas are defined as areas in the pathological slide where there is no discrete presence of tumor cells. Within the areas annotated by a pathologist, we selected regions of interest (ROI) that were subsequently digitized using HS instrumentation. Within each ROI, different numbers of HS images were acquired for analysis. Figure 2 shows an example of the annotations within the pathological slide, the selection of different ROIs (shown at 5×), and the HS images (imaged at 20×) that are used in this study for classification. In this case, red color annotations indicate areas diagnosed as GB, while non-tumor areas were annotated in blue marker. In this feasibility study, a total of 13 patients were analyzed.

### 2.3. Hyperspectral Dataset

**Figure 3:**
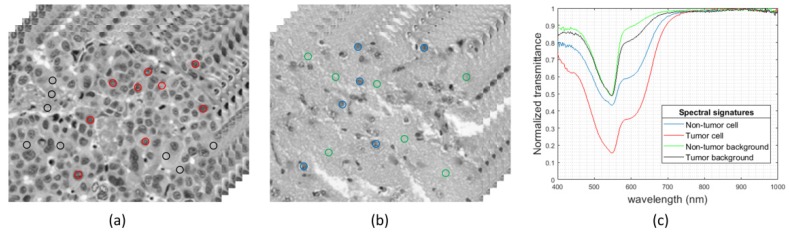
HS histopathological dataset. (**a**,**b**) HS cubes from tumor and non-tumor samples, respectively. (**c**) Spectral signatures of different parts of the tissue: tumor cells (red), non-tumor cells (blue), tumor background tissue (black), and non-tumor background tissue (green).

Using the aforementioned instrumentation, some of the areas highlighted by pathologists from each slide were imaged. The positioning joystick of the microscope was used to select the initial position of the first HS image within a ROI to be captured. Then, we configured in the software the number of images to be captured consecutively. This number of images should keep relatively low to avoid the focus worsening of the images throughout the specimen. In this case, a maximum of 10 HS images were extracted consecutively from a ROI. We used a 20× magnification for image acquisition, producing a HS image size of 375 × 299 µm. This magnification was chosen because it allowed the visualization of the cell morphology; hence, the classifier was able to exploit both the spatial and the spectral features of data. In Figure 3, we show some examples of HS images used in this study, together with the spectral signatures of representative tissue components, i.e., cells and background for both tumor and non-tumor regions.

In this research, we used a CNN to perform the classification of the samples. Due to the nature of the data for this study, the ground truth assignment into tumor or non-tumor is shared across each selected ROI; thus, each HS image is assigned within a certain class. For this reason, it was decided to perform the classification in a patch-based approach because a fully-convolutional design was not feasible. There are two motivations on the selection of the patch size. Firstly, the patch should be large enough to contain more than one cell, but if the patch is too large, then the CNN could learn that the tumor is located only in dense cell patches. Secondly, the smaller the patches, the higher the quantity of patches will be extracted from a single HS image, so the number of samples to train the CNN will be increased. Finally, we choose a patch size of 87 × 87 pixels. In principle, from a spectral cube of size 800 × 1004, 99 patches can be extracted. However, there are some situations where most parts of the patches consisted only of a blank space of light. For this reason, we decided to reject patches that were composed by more than 50% of light, i.e., half of the patch is empty.

**Figure 4:**
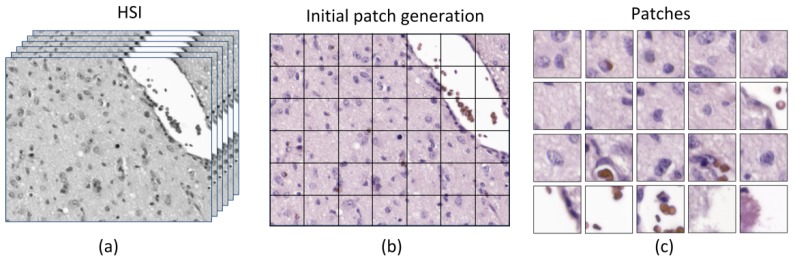
Generation of patches. (**a**) Original HS image; (**b**) grid of patches within the HS image; (**c**) patches of size 87 × 87 used in the classification. The last row contained patches that were rejected for the dataset for having more than 50% of empty pixels. HSI: hyperspectral imaging.

The method to reject the patches which presented high amount of light is as follows. Firstly, the RGB image is extracted from the HS cube and is transformed to the hue-saturation-value color representation. Then, the hue value of each image is extracted and binarized using a threshold empirically configured to separate the pixels belonging to the specimen and the pixels containing background light. The generation of the patches can be observed in Figure 4, where the last row of the patches (in Figure 4c) represents patches that have been rejected in the database due to high content of background light pixels.

The database used in this work consists of 527 HS images, where 337 are non-tumor brain samples and 190 were diagnosed as GB. It should be highlighted that only the biopsies from 8 patients presented both non-tumor and tumor samples; the other 5 patients only presented tumor samples. The summary of the employed dataset is detailed in Table 1. After extracting the patches that were valid to be processed, we had a total of 32,878 patches from non-tumor tissue and 16,687 from tumor tissue.

### 2.4. Processing Framework

The processing framework applied to each HS cube is composed by the following steps. First, a standard flat field correction is applied to the images. To this end, the images are transformed from radiance to normalized transmittance by using a reference image that is captured from a blank area of the pathological slide [23]. Then, due to the high correlation of spectral information between adjacent spectral bands, a reduced-band HS image is generated by averaging the neighbors’ spectral bands, reducing the number of spectral bands from 826 bands to 275 and slightly reducing the white Gaussian noise. This band reduction is also beneficial for alleviating computational cost in the subsequent image processing. Finally, each image is divided into patches, which will train the CNN. In this section, we will detail the architecture of the proposed neural network, the metrics that are used for performance evaluation, and the proposed data partition scheme.

#### 2.4.1. Convolutional Neural Network

We employed a custom 2D-CNN for the automatic detection of non-tumor and tumor patches. As mentioned previously, these types of networks are able to exploit together the spatial and spectral features of the sample. The performance of DL approaches for the classification of HS data has been proven both for medical and for non-medical applications [26]. We used the TensorFlow implementation of the Keras Deep Learning API [27,28] for the development of this network. This selection was made because it allows effective development of CNN architectures, training paradigms, and efficient deployment between the Python programming language and GPU deployment of training/testing. The architecture of this CNN is mainly composed by 2D convolutional layers. We detail the description of the network in Table 2, where the input size of each layer is shown in each row, and the output size is the input size of the subsequent layer. All convolutions and the dense layer were performed with ReLU (rectified linear unit) activation functions with a 10% dropout. The optimizer used was stochastic gradient descend with a learning rate of $10^{-3}$.

#### 2.4.2. Evaluation Metrics

The metrics for measuring the classification performance of the proposed CNN were overall accuracy, sensitivity, and specificity. Overall accuracy measures the overall performance of the classification; sensitivity measures the proportion of true positives that are classified correctly; and specificity measures the ability of the classifier for identifying false negatives. The equations for these metrics according to the false positives (FP), false negatives (FN), true positives (TN), and true negatives (TN) are shown in Equations (1)−(3). Additionally, we used the area under the curve (AUC) of the receiver operating curve (ROC) of the classifier as an evaluation metric. The AUC has been proven to be more robust compared to overall accuracy. AUC is decision threshold independent, shows a decreasing standard error when the number of test samples increases, and is more sensitive to Analysis of Variance (ANOVA) test [29].

(1) $$Sensitivity=\frac{TP}{TP+FN},$$

(2) $$Specificity=\frac{TN}{TN+FP},$$

(3) $$Accuracy=\frac{TN+TP}{TN+TP+FP+FN}.$$

#### 2.4.3. Data Partition

In this work, we split data into training, validation and test sets. We were targeting a real clinical application, and, for this reason, data partition is intended to minimize bias, where the patients used for train, validation and test are independent. We were limited to 13 patients, where five of them only had samples belonging to tumor class. For this reason, we decided to perform the data partition in 4 different folds, where every patient should be part of the test set across all the folds. We proposed the use of three folds with 9 training patients, a single validation patient, and 3 test patients. The remaining fold is composed by 8 training patients, a single validation patient, and 4 test patients. Regarding the distribution of the classes in each fold, the patient selected for validation in each fold should have samples from both types of classes (non-tumor and tumor). The initial data partition scheme is shown in Table 3, where data from patients who only have tumor samples has been highlighted (^‡^).

We decided to make the patient assignment randomly within the different folds. However, the distribution of patients in fold F4 was different from the others and required some minor manual adjustments in data partitioning. Nonetheless, the rest of assignments were performed randomly. Fold F4 required assigning two tumor-only specimens for testing, so we decided to manually assign the tumor-only samples that have the least number of patches (i.e., P10 and P12). Furthermore, because fold F4 had fewer training patients compared to the other folds, we decided to assign the patient with the most patches (i.e., P5) to train this fold. The final data partition into the different folds is shown in Table 4.

## 3. Experimental Results

### 3.1. Validation Results

We trained different CNNs using the data from each fold, and, using the validation data, we selected the aforementioned CNN architecture (Table 2) as the best candidate for the classification of the samples. As can be observed in Table 1, the data between tumor and non-tumor classes are not balanced: the number of non-tumor samples is twice the number of tumor samples. For this reason, we performed data augmentation on the tumor data to balance the data during training, creating twice the number of tumor patches to train the CNN than cited in Table 1. Such data augmentation consisted in a single spatial rotation of tumor patches.

At the beginning of the validation phase, some of the folds presented problems when they were trained, showing poor performance metrics in the validation set. For this reason, we carefully examined the tumor HS images from each patient, and we detected that accidentally some necrosis areas were included in the dataset as tumor samples. These necrosis areas (found in P8) were excluded from the dataset. After excluding the necrosis areas, we got competitive results for all the folds in the validation set. These results are shown in Table 5. The models for each fold were selected because they all presented high AUC, higher than 0.92, and the results in terms of accuracy, sensitivity and specificity were balanced, indicating that the models identified correctly both non-tumor and tumor tissue.

In order to provide a comparison of performance between HSI and RGB imagery, we performed the classification of synthetic RGB images using the same CNN. Such RGB images were extracted from the HS data, where each color channel was generated equalizing the spectral information to match the spectral response of the human eye [30]. After separately training the CNN with RGB patches, the models selected after the validation were found to be competitive. Nevertheless, the validation performance when using HSI data was more accurate in each fold and presented more balanced sensitivity and specificity values (Table 5).

### 3.2. Test Results

After the model selection in the validation phase, we applied them to independent patients for the test set. These results are shown in Table 6. Some results show good discrimination between non-tumor and tumor tissues, i.e., patients P1, P3, and P8. For these patients, the AUC, sensitivity and specificity are comparable to the values obtained during validation. The tumor detection in patients P9 to P13 was also highly accurate. However, there are some patients where the classification performance was poor. Although the sensitivity is high in patients P2 and P5, the specificity is low, which indicates there may be an issue classifying non-tumor patches. There are also some patients with poor accuracy, namely patients P4 and P7, which have results slightly better than random guessing. Finally, the results obtained for patient P6 are suspicious, being substantially worse than random guessing.

**Figure 5:**
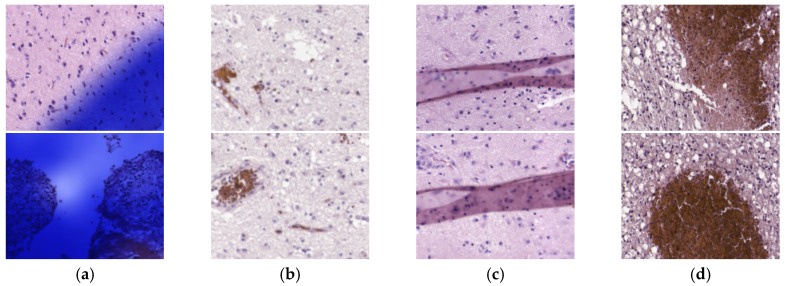
Example of image defects detected in the test dataset. (**a**) Ink contamination; (**b**) unfocused images; (**c**) artifacts in the specimens; (**d**) samples mainly composed of red blood cells.

The selection of the models in the validation phase was performed using independent patients for validation; for this reason, such inaccuracies on test data was unexpected. To determine the reasons for the misclassifications, we used the CNN models to generate heat maps for all the patients, and we carefully examined them. After this analysis, we found that some HS images presented problems; hence, the results were worsened for these reasons. As mentioned before, we performed a careful inspection of tumor HS images in the validation set. However, upon inspection after the test outcomes, we discovered there were also problems in non-tumor samples. There were four main sources of errors in the images: (1) some HS images were contaminated with the ink used by pathologists to delimitate the diagnosed regions (n=15); (2) some images were unfocused (n=13); (3) some samples presented artifacts from histopathological processing (n=2); and (4) other images were composed mainly by red blood cells (n=2). Examples of these images can be observed in Figure 5.

**Figure 6:**
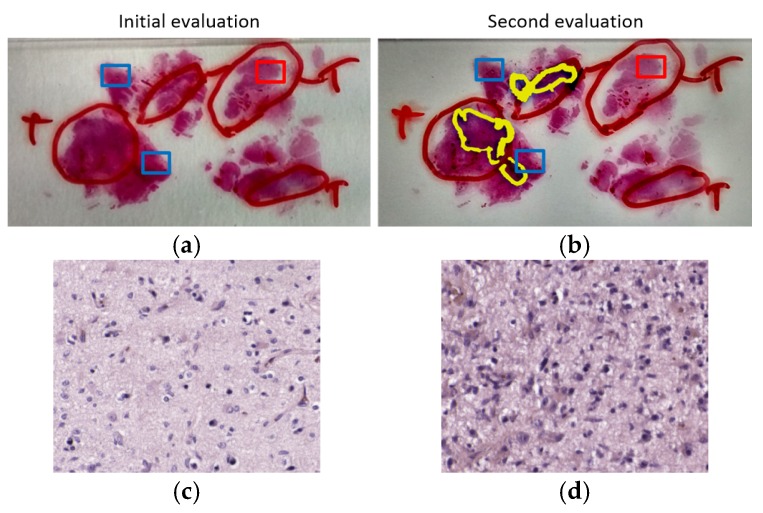
Evaluation assessment for the samples of Patient P6. Red pen markers indicate the initial evaluation of tumor regions. Regions without pen contour were considered as non-tumor. Red squares indicate the ROIs of tumor samples. Blue squares indicate the ROIs of non-tumor samples. (**a**) Initial evaluation of the sample; (**b**) second evaluation of the sample, where a yellow marker is used for the updated tumor areas; (**c**) example of HSI from tumor ROI; (**d**) example of HSI from non-tumor ROI.

Furthermore, due to the suspicious results obtained on patient P6, the specimen was examined again by a pathologist for reassessing the initial diagnosis. After this examination, the pathologist realized a problem with the selection of ROIs in the HS acquisition for the non-tumor areas. In Figure 6, we show the initial evaluation of the sample, where the tumor area was annotated by using a red marker contour, and the rest of the sample was considered as non-tumor. Figure 6b corresponds to the second evaluation of the sample. The original annotation of tumor was technically correct, but the yellow markers indicate the location of the highly invasive malignant tumor, i.e., GB. Although the other tumor areas correspond to tumor, their cells are atypical and cannot be considered a high-grade GB. In both Figure 6a,b, the ROIs selected for HS acquisitions are highlighted with squared boxes, where red and blue boxes indicate tumor and non-tumor ROIs, respectively. As can be observed in Figure 6b, the non-tumor areas selected for our experiments were located too close to areas where the infiltrating GB was identified; thus, they contain extensive lymphocytic infiltration and cannot be considered strictly non-tumor samples. Furthermore, it was found that the GB of this patient was not typical, presenting low cellular density in the tumor areas. Finally, the ROI selected from the tumor area was located where the diagnosis is tumor but cannot be considered a high-grade glioma, i.e., GB. These reasons explain the seemingly inaccurate results obtained in the classification. Nevertheless, such bad results helped us to find an abnormality in the sample.

In order to quantify the influence of the inclusion of incorrect HS images in the classification, we evaluated again the classifiers when the corrupted HS images were excluded from the dataset. These HS images were only removed from the test. The CNN was not trained again to avoid introducing bias in our experiments. These results are shown in Table 7. Patients where data exclusion was performed are indicated with an asterisk (*), and the results of patient P6 were removed due to the diagnosis reasons explained before. The results of the classification after data exclusion improved significantly for patients P2 and P7, while the results of other patients keep constant after the exclusion of some HS images. This data removal also boosts the overall metrics across the patients, due to the improvement in the classification in some patients and because of the removal of patient P6 due to justifiable clinical reasons.

Regarding the classification performance of HSI compared to RGB, the results suggest the superiority of HSI (see Table 6 and Table 7). The average metrics on the whole datasets are worse for RGB images, especially in terms of specificity and sensitivity. We consider good performance in classification when all the metrics are high, with balanced specificity and sensitivity. For example, P4 presents a better AUC for RGB but really poor specificity (7%). For this reason, the HSI classification for such patient presents a better performance. Only for P2, P3, and P8, the performance of RGB is approximately equivalent to HSI. P11 is the only patient where RGB substantially outperforms HSI. For patients where the performance is the most promising (e.g., P1, P2, P3, and P8), RGB classification is also accurate. However, the sensitivity and specificity are not as balanced compared to HSI. Furthermore, the standard deviation in specificity and sensitivity are higher for RGB classification, which show a wider spread of the classification results compared to HSI. The decrease of performance of RGB images compared to HSI is more evident in patients with only tumor samples, where HSI classification was shown to be really accurate (e.g., P9, P10, P12, and P13). Finally, in patients where the classification of HSI was found poor (e.g., P4, P5, and P7), HSI performance is still shown to be more competitive than the RGB counterpart. On average, the accuracy of the classification is improved 5% when using HSI instead of RGB imaging, and particularly, the specificity and specificity are increased achieving 7% and 9% of improvement, respectively (Table 7).

### 3.3. Heat Map Results

**Figure 7:**
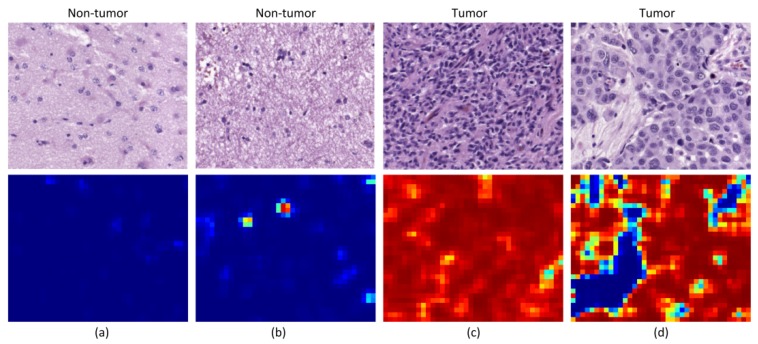
Heat maps from good performance patients. (**a**) Non-tumor tissue with no false positive; (**b**) non-tumor tissue with some false positives; (**c**) tumor tissue with no false negative; (**d**) tumor tissue with some false negatives.

**Figure 8:**
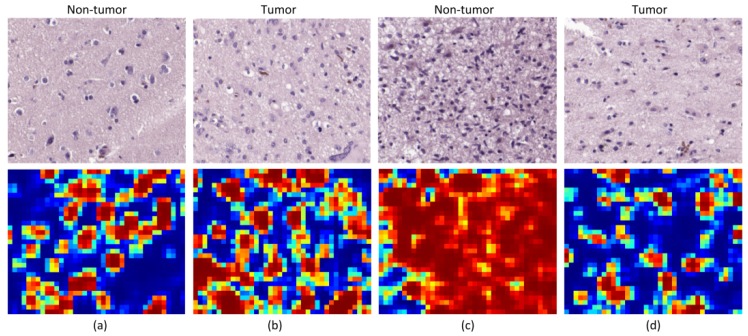
Heat maps from bad performing patients. (**a**,**b**) Non-tumor and tumor maps from Patient P4; (**c**,**d**) non-tumor and tumor maps from Patient P6.

Beyond the results obtained for the analysis of the patches, we also qualitatively evaluated the outcomes of the classification by generating classification heat maps from the HS images. In these maps, the probability of each pixel to be classified as tumor is represented, where red values indicate high probability and blue values indicate low probability. The inputs of our CNN are patches of 87 × 87 pixels, for this reason the resolution of the heat maps cannot contain pixel-level details. To provide them with resolution enough for a useful interpretation, we generated classification results for a 23-pixels sliding window length. We show two different types of heat maps in Figure 7 and Figure 8.

On the one hand, in Figure 7, we illustrate the different types of results that are obtained in patients where the models were proven to classify accurately the samples. Figure 7a,c show examples of non-tumor and tumor images that were classified correctly, with no presence of neither false positives nor false negatives, respectively. In Figure 7b, we can see the presence of some false positives in a non-tumor tissue image, but such false positives are located in an area where there is a cluster of cells, indicating that it is a suspicious region. Finally, Figure 7d shows a tumor image where there are some regions classified as non-tumor tissue. Nevertheless, such false negatives are located in areas where there are no cells. The FP in Figure 7b suggests the CNN perceives areas with high cell density as tumor. The FN shown in Figure 7d has a clinical interpretation, but it is computed as a bad result in the quantitative evaluation of the classification. Furthermore, a more detailed ground truth scheme for classification may improve the classification performance, e.g., the inclusion of brain background tissue, blood vessels, or blood cells.

On the other hand, we show, in Figure 8, the heat maps from patients that present the worst performance in the quantitative evaluation of the results. Firstly, Figure 8a,b show the results for Patient P4. It can be observed that the heat maps for each kind of tissue are similar, presenting false positives for the non-tumor image and false negatives for the tumor image. For this patient, the heat maps and the quantitative results are coherent, showing that the CNN is not able to accurately classify the samples from this patient. Secondly, Figure 8c,d show the heat maps for Patient P6. As mentioned before, the non-tumor tissue of this patient was proven to be adjacent to the tumor area, and hence cannot be considered as non-tumor tissue. In this case, Figure 8c shows that the non-tumor area has been classified as tumor, which is in fact correct. Finally, it was also discussed that Patient P6 presented a non-typical GB with low cellularity. A heat map from a tumor image from this patient (Figure 8d) shows that tumor cells are highlighted as tumor, but the areas with low cellular presence are diagnosed as non-tumor.

## 4. Discussion and Conclusion

In this research work, we present a hyperspectral microscopic imaging system and a deep learning approach for the classification of hyperspectral images of H&E pathological slides of brain tissue samples with glioblastoma of human patients.

As described in the introduction, this research is the continuation of a previous research that presented some drawbacks [23]. Firstly, the total number of HS cubes used in our previous work was limited to 40, only 4 HS cubes per patient. Secondly, the instrumentation used in this previous work presented limitations in both the spectral and spatial information. Regarding the spectral information, the spectral range was restricted to 419–768 nm due to limitations of the microscope optical path. The spatial information was limited due to the use of a scanning platform unable to image the complete scene, so the analysis of the HS images was restricted to a low magnification (5×), which was not sufficient to image the morphological features of the sample. Additionally, the main goal of such previous work was to develop a preliminary proof-of-concept on the use of HSI for the differentiation of tumor and non-tumor samples, showing promising results.

In this work, an improved acquisition system capable of capturing high-quality images in a higher magnification (20×), and with a higher spectral range (400–1000 nm) has been used to capture a total amount of 517 HS cubes. The use of 20× magnification allows the classifier to exploit both the spectral and the spatial differences of the samples to make a decision.

Such dataset was then used to train a CNN and to perform the classification between non-tumor and tumor tissue. Due to a limited number of patients involved in this study and with the aim to provide a data partition scheme with minimum bias, we decided to split the dataset in four different folds where the training, validation, and testing data belonged to different patients. Each fold was trained with 9 patients, where only 5 of them presented both types of samples, i.e., tumor and non-tumor tissue.

After selecting models with high AUC and balanced accuracy, sensitivity and specificity in the validation phase, some results on the test set were not accurate at all. For this reason, we carefully inspected the heat maps generated by the classifiers for each patient in order to find a rationale about the inaccurate results. After this, we detected four types of problems in the images that could worsened the results, namely the presence of ink or artifacts in the images, unfocused images, or excess of red blood cells. We reported both results, before and after cleaning wrong HS images, for a fair experimental design. We consider that the test results after removing such defective HS images are not biased because the rationale of removing the images from the test set is justifiable and transparent. These corrupted images were part of the training set, but it is unknown if the training process of the CNN was affected.

We also found a patient, P6, where the results were really inaccurate. For this reason, the regions of interest that were analyzed by HS were re-examined by the pathologists. After examining the sample, an atypical subtype of GB was found, and examination revealed that the ROIs selected as normal samples were close to the tumor area, which cannot be considered as non-tumor. Although the classification results were not valid for this patient, by using the outcomes of the CNN, we were able to identify a problem with the prior examination and ROI selection within the sample. Additionally, although this patient was used both as part of the training and as a patient used for validation, the results are not significantly affected by this fact. These results highlight the robustness of the CNN for tumor classification. Firstly, although the validation results of fold 1 were good when evaluating patient P6, the model from this fold was also capable of accurately classifying patients P1 and P11. Secondly, although patient P6 was used as training data for fold 3 and fold 4, the outcomes of these models were not proven to be significantly affected by contaminated training data.

Although the results are not accurate in every patient, after excluding incorrectly labeled and contaminated HS images, nine patients showed accurate classification results (P1 to P3 and P8 to P13). Two patients provided acceptable results (P5 and P7), and only a single patient presented results that were slightly better than random guessing (P4). Nevertheless, these results can be considered promising for two main reasons. First, a limited number of samples were used for training, especially for the non-tumor class, which was limited to only five training patients for each CNN. Second, the high inter-patient variability shows significant differences between tumor samples among the different patients. As can be observed in the analysis of heat maps (Figure 7 and Figure 8), there is a significant heterogeneity in cellular morphology in different patients’ specimens, which makes GB detection an especially challenging application. To handle these challenges, the number of patients should be increased in future works, and to deal with the high inter-patient variability, HS data from more than a single patient should be used to validate the models.

Finally, we found that HSI data perform slightly better than RGB images for the classification. Such improvement is more evident when the classification is performed on challenging patients (e.g., P5 or P7) or in patients with only tumor samples. Furthermore, the classification results of HSI are shown to provide more balanced sensitivity and specificity, which is the goal for clinical applications, improving the average sensitivity and specificity by 7% and 9% with respect to the RGB imaging results, respectively. Nevertheless, more research should be performed to definitively demonstrate the superiority of HSI over conventional RGB imagery.

## Figures and Tables

**Table 1:** HS histopathological dataset summary.

Patient ID	Images		Patches	
	Non-Tumor	Tumor	Non-Tumor	Tumor
P1	48	12	4595	1090
P2	36	12	3563	1188
P3	31	12	3058	1178
P4	40	12	3779	1158
P5	66	12	5675	1165
P6	48	12	4586	1188
P7	44	12	4289	1184
P8	24	36	3333	2260
P9	0	22	0	1695
P10	0	12	0	1094
P11	0	12	0	1169
P12	0	12	0	1137
P13	0	12	0	1181
Total	337	190	32,878	16,687

**Table 2:** Schematic of the proposed convolutional neural network (CNN).

Layer	Kernel Size	Input Size
Conv2D	3 × 3	87 × 87 × 275
Conv2D	3 × 3	85 × 85 × 256
Conv2D	3 × 3	83 × 83 × 256
Conv2D	3 × 3	81 × 81 × 512
Conv2D	3 × 3	79 × 79 × 512
Conv2D	3 × 3	77 × 77 × 1024
Conv2D	3 × 3	75 × 75 × 1024
Conv2D	3 × 3	73 × 73 × 1024
Global Avg. Pool	25 × 25	73 × 73 × 1024
Dense	256 neurons	1 x 1024
Dense	Logits	1 × 256
Softmax	Classifier	1 × 2

**Table 3:** Data partition design (patients with only tumor samples are marked with ‡).

Fold ID	Training Patients	Validation Patients	Test Patients
F1	9 (5 + 4^‡^)	1	3 (2 + 1^‡^)
F2	9 (5 + 4^‡^)	1	3 (2 + 1^‡^)
F3	9 (5 + 4^‡^)	1	3 (2 + 1^‡^)
F4	8 (5 + 3^‡^)	1	4 (2 + 2^‡^)

**Table 4:** Final data partition (patients with only tumor samples are marked with ‡).

Fold ID	Training Patients	Validation Patients	Test Patients
F1	P2, P3, P4, P5, P8	P6	P1, P7, P11^‡^
	P9^‡^, P10^‡^, P12^‡^, P13^‡^		
F2	P1, P2, P5, P7, P8	P3	P4, P6, P13^‡^
	P9^‡^, P10^‡^, P11^‡^, P12^‡^		
F3	P1, P3, P4, P6, P8	P7	P2, P5, P9^‡^
	P10^‡^, P11^‡^, P12^‡^, P13^‡^		
F4	P2, P4, P5, P6, P7	P1	P3, P8, P10^‡^, P12^‡^
	P9^‡^, P11^‡^, P13^‡^		

**Table 5:** Classification results on the validation dataset, across all four folds (F). AUC: area under the curve.

Partition	HSI				RGB			
	AUC	Accuracy (%)	Sensitivity (%)	Specificity (%)	AUC	Accuracy (%)	Sensitivity (%)	Specificity (%)
F1	0.92	84	84	85	0.88	77	71	88
F2	0.97	93	91	94	0.95	87	83	93
F3	0.95	88	90	88	0.93	87	91	79
F4	0.95	89	87	91	0.92	92	93	89
Avg.	0.95	88	88	89	0.92	86	84	87
Std.	0.02	3.70	3.16	3.87	0.03	6.29	9.98	5.91

**Table 6:** Initial classification results on the test dataset.

Patient ID	HSI				RGB			
	AUC	Accuracy (%)	Sensitivity (%)	Specificity (%)	AUC	Accuracy (%)	Sensitivity (%)	Specificity (%)
P1	0.97	92	90	94	0.92	90	97	61
P2	0.75	77	99	69	0.98	85	99	80
P3	0.95	85	91	80	0.96	92	97	78
P4	0.62	57	57	58	0.69	77	98	7
P5	0.81	69	81	64	0.66	59	59	60
P6	0.35	37	38	36	0.21	67	81	7
P7	0.64	59	64	57	0.51	45	36	76
P8	0.98	96	96	96	0.99	97	97	97
P9	N.A.	99	99	N.A.	N.A.	89	89	N.A.
P10	N.A.	89	89	N.A.	N.A.	43	43	N.A.
P11	N.A.	92	92	N.A.	N.A.	98	98	N.A.
P12	N.A.	92	92	N.A.	N.A.	84	84	N.A.
P13	N.A.	99	99	N.A.	N.A.	88	88	N.A.
Avg.	0.76	80	84	69	0.74	78	82	58
Std.	0.22	19	19	20	0.28	19	22	34

**Table 7:** Final classification results on the test set after excluding incorrect HS images.

Patient	HSI				RGB			
	AUC	Accuracy (%)	Sensitivity (%)	Specificity (%)	AUC	Accuracy (%)	Sensitivity (%)	Specificity (%)
P1*	0.98	93	91	96	0.93	90	97	61
P2*	0.99	89	99	83	0.99	87	79	99
P3*	0.95	85	91	80	0.96	92	97	78
P4	0.62	57	57	58	0.69	77	98	7
P5*	0.81	69	81	64	0.66	58	57	60
P6^†^	-	-	-	-	-	-	-	-
P7*	0.74	66	71	63	0.68	58	50	77
P8	0.98	96	96	96	0.99	97	97	97
P9	N.A.	99	99	N.A.	N.A.	89	89	N.A.
P10	N.A.	89	89	N.A.	N.A.	43	43	N.A.
P11	N.A.	92	92	N.A.	N.A.	98	98	N.A.
P12	N.A.	92	92	N.A.	N.A.	84	84	N.A.
P13	N.A.	99	99	N.A.	N.A.	88	88	N.A.
Avg.	0.87	85	88	77	0.84	80	81	68
Std.	0.15	14	13	16	0.16	18	20	31

* Data exclusion; ^†^ Data removed.
